# Supplementary material for: Acute Modification of Hemodynamic Forces in Patients with Severe Aortic Stenosis after Transcatheter Aortic Valve Implantation
Source: J Clin Med. 2023 Feb 3;12(3):1218. doi: 10.3390/jcm12031218 (PMC9917967; doi:10.3390/jcm12031218)
Supplement: Supplementary file 1 [file jcm-12-01218-s001.zip › jcm-2164895-supplementary.pdf]

Acute modification of Hemodynamic Forces in patients with severe aortic stenosis after Transcatheter Aortic Valve Implantation

Supplementary Material

Physical definition of HDFs

The intracardiac blood flow direction is driven by the pressure gradient between chambers, from regions with a higher pressure to those at a lower pressure, with competent valves preventing retrograde flow.

HDFs represents the global force exchanged between blood volume and endocardium. It is derived from the summation of the individual intraventricular pressure gradients (IVPGs) driving blood inside the ventricular chamber of interest. This global force, by the relationship between pressure gradient and velocity field (Navier-Stokes equation), is

$$F(t) = \rho \int_{V(t)} \left(\frac{\partial v}{\partial t} + v \cdot \nabla v \right) dV, \qquad \text{(S1)}$$

where v (x, t) is the blood velocity at any x point inside the LV volume V(t), at any instant of time and ρ is a constant expression of the blood density (ρ =1.05 kg/L).

The HDFs force vector, F(t), is directed from the highest to the lowest pressure area. The intraventricular HDFs direction may not always coincide with blood flow direction, because HDFs aligns with the direction of the acceleration of blood flow that, in unsteady motion, may require some time to modify its direction after the application of force.

Using the blood velocity recorded in the LV pool with 4D flow MR, the formula (S1) allows the estimation of the HDFs. The same force vector can also be computed from an integral over the LV boundary, S(t), instead of the internal volume.

$$F(t) = \rho \int_{S(t)} \left[x \left(\frac{\partial v}{\partial t} \cdot n \right) + v (v \cdot n) \right] dS. \qquad \text{(S2)}$$

This formula requires the knowledge of the velocity at the endocardial edge and across the valves and does not require measuring the blood velocity inside the whole ventricular

cavity. Therefore, it allows evaluation of HDFs by measuring the moving ventricular geometry and the valve orifice and can be employed with standard multiplane or 3D echocardiographic exams, without the need of a 4D flow MR system. Although formula (S2) may appear complex, it corresponds to the integration of values given by tissue position, velocity, and acceleration at the endocardial border. These values are commonly used in other calculations, such as strain and strain rate. Similarly, the average blood velocity across the valve can be evaluated from the volumetric changes using the principle of mass conservation. Therefore, the HDFs estimation from formula (S2) can be included in currently available imaging solutions that compute volumetric rates or are dedicated to deformation imaging. Subsequently, it can be applied to standard images recorded during an echocardiographic examination.

HDFs in LV occur along 3 planes: basal-apical, septal-lateral, and inferior-anterior. HDFs in the right ventricle occur along the diaphragm-outflow tract, basal-apical and septal-free wall directions[1].

The basal-apical direction is the most widely reproducible and detectable force in all patients. When the HDFs vector is directed from the apex to base of LV (when apical pressure is higher than basal pressure), it is defined as a positive deflection (above the zero line); when the HDFs vector is directed from the base to apex (basal pressure higher than apical pressure), it is defined as a negative deflection (below the zero line).

Supplementary References

1. Töger J, Arvidsson PM, Bock J, Kanski M, Pedrizzetti G, Carlsson M, *et al.* Hemodynamic forces in the left and right ventricles of the human heart using 4D flow magnetic resonance imaging: Phantom validation, reproducibility, sensitivity to respiratory gating and free analysis software. *PLoS One* PLoS One; 2018;**13**.
